# Long COVID in Inflammatory Bowel Diseases

**DOI:** 10.3390/jcm10235575

**Published:** 2021-11-26

**Authors:** Silvia Salvatori, Francesco Baldassarre, Michelangela Mossa, Giovanni Monteleone

**Affiliations:** 1 Gastroenterology Unit, Department of Systems Medicine, University of Rome Tor Vergata, 00133 Rome, Italy; silviasalvatori23@gmail.com (S.S.); michelangela.mossa@gmail.com (M.M.); 2 Department of Systems Medicine, University of Rome Tor Vergata, 00133 Rome, Italy; francesco.baldassarre96@gmail.com

**Keywords:** Crohn’s disease, ulcerative colitis, SARS-CoV-2, biologics

## Abstract

Background and aims. SARS-CoV-2-infected patients can experience long-lasting symptoms even after the resolution of the acute infection. This condition, defined as Long COVID, is now recognized as a public health priority and its negative impact on the quality of life of the patients could be more relevant in individuals with debilitating pathologies. We here evaluated the frequency of Long COVID in patients with inflammatory bowel diseases (IBD). Methods. IBD patients afferent for scheduled visits to our tertiary referral center at the Tor Vergata University Hospital, Rome, were recruited from 7 September to 22 October 2021. During the visits, patients were investigated about previous COVID-19 infection and the possible development of Long COVID. Results. Fifty-three out of 528 IBD patients (10%) have had a SARS-CoV-2 infection. Of these, 21 patients (40%) developed Long COVID, and asthenia was the more frequent symptom as it occurred in nearly two-thirds of patients. Patients with Long COVID were more frequently females, while other clinical and demographic characteristics did not differ between patients with Long COVID and those without Long COVID. In particular, the IBD relapses occurred with the same frequency in the two groups. Conclusions. Long COVID appears to be common in IBD patients even though it does not influence the IBD course.

## 1. Introduction

From December 2019, after the first appearance of Severe Acute Respiratory Syndrome 2 Coronavirus (SARS-CoV-2) in Wuhan, China, we witnessed the onset of a pandemic, which represents the biggest health and economic challenge of the last 100 years [1,2]. For this reason, unprecedented mass vaccination is underway worldwide, and at the moment, nearly half of the world population has received at least one dose of a COVID-19 vaccine (https://ourworldindata.org/coronavirus, Accessed on 2 November 2021). In the last two years, our knowledge about biological and clinical features of COVID-19, the disease caused by SARS-CoV-2, has increased [3], but there are still many characteristics of this new nosological entity to clarify. The most common clinical presentation of COVID-19 is a mild respiratory infection and, less commonly, pneumonia with fever, cough, and dyspnoea and other signs and symptoms, such as odynophagia, headaches, anosmia, ageusia, myalgia, and diarrhea [4,5,6]. SARS-CoV-2 enters in cells binding ACE2 receptor, which is expressed by numerous cell types throughout the human body. The infection causes an inflammatory response in multiple organs, thereby explaining the extreme variability of clinical manifestations [7]. It is now evident that some patients may experience signs and symptoms for several months after the resolution of SARS-CoV-2 infection, a condition which has been termed ‘‘Long COVID,’’ ‘‘COVID-19 long-haulers,’’ or post-acute COVID-19 syndrome [8]. Manifestations of Long COVID are clinically diverse and supposed to be caused by multiple underlying mechanisms [9]. In particular, it has been hypothesized that persistent systemic immune activation and inflammation may contribute to the Long COVID pathogenesis, but at the present time, it remains unclear if this condition occurs more frequently in patients with coexisting inflammatory pathologies. The latter category could theoretically include patients taking immunomodulatory compounds, as these drugs could attenuate the SARS-CoV-2 immunity.

Inflammatory bowel diseases (IBD) are chronic, multifactorial inflammatory disorders of unknown etiology, sustained by an exaggerated and poorly controlled immune response against luminal antigens [10]. Indeed, drugs blocking immune cell function are used with success for inducing and maintaining remission in patients with Crohn’s disease (CD) and patients with ulcerative colitis (UC), the major IBD in human beings [11]. Unfortunately, however, the use of these therapies has been associated with an increased risk for bacterial and viral infections and viral reactivation [12]. Several authors have investigated the epidemiology, clinical characteristics, and outcomes in IBD patients with COVID-19, and a recent systematic review and meta-analysis of 14 studies including 50706 IBD patients with and without COVID-19 reported a low prevalence of COVID-19 in IBD patients and a worse outcome of COVID-19 in IBD patients receiving corticosteroids or mesalamine, while the use of anti-TNFs was associated with more favorable outcomes [13]. In contrast, there is no data about the prevalence and impact of Long COVID in IBD. 

In the present study, we examined the frequency of symptoms/signs suggestive of Long COVID in IBD and assessed possible risk factors for this condition. 

## 2. Methods

This was an observational study including IBD patients regularly followed in our tertiary referral center at the “Tor Vergata University Hospital”, Rome, Italy. During the scheduled follow-up visits, demographic and clinical characteristics of the patients (i.e., sex, age, current therapy, and relapses) were collected. Moreover, we collected data about previous COVID-19 infection (i.e., rhino-pharyngeal swab positive for SARS-CoV-2), symptoms and duration of, and hospitalization and death due to COVID-19. In SARS-CoV-2-infected patients, we also collected information regarding the persistence of symptoms for at least four weeks or the onset of new ones after the resolution of infection [8]. All patients provided their informed consent for the use of personal and clinical information for scientific purposes, and no one refused to participate. 

Categorical study variables were expressed as number and proportion (%), and continuous variables were expressed as median [range]. Patients’ characteristics were compared using the χ ^2^ for the categorical variables and the Student’s *t*-test for continuous variables. Statistical analyses were performed using IBM SPSS Statistics 27.0.1.0. 

## 3. Results

**Figure 1:**
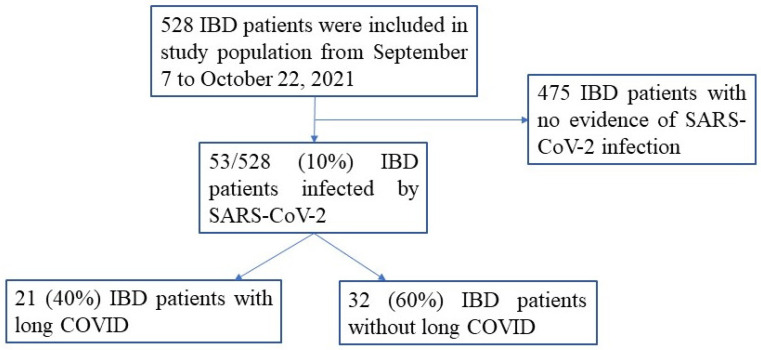
Flow diagram of the inflammatory bowel disease (IBD) patients included in the study.

From 7 September to 22 October 2021, we collected information of 528 IBD patients afferent to our center for scheduled follow-up visits (Figure 1). COVID-19 duration was defined as the time between the first SARS-CoV-2 positive rhino-pharyngeal swab and the first negative swab. Fifty-three out of 528 patients (10%) have had a SARS-CoV-2 infection, which caused disparate symptoms and signs, hospitalization in only four patients, but no death (Table 1). In all the cases, infection occurred before SARS-CoV-2 vaccine administration, and only 44 of them (83%) received vaccination after SARS-CoV-2 infection resolution. Twenty-one out of 53 patients (40%) reported persistence of symptoms for more than four weeks after the resolution of SARS-CoV-2 infection, so they were classified as Long COVID carriers (Table 2). Asthenia was the more frequent symptom as this occurred in nearly two-thirds of the patients for a median period of six months (Table 2). Additional persistent symptoms included ageusia, anosmia, headache, asthenia, and dyspnea, while the remained symptoms appeared just after SARS-CoV-2 infection resolution. Comparison of clinical and demographic characteristics between patients with Long COVID and those without Long COVID demonstrated that patients with Long COVID were more frequently females. No other significant difference was seen between the groups (Table 1). In particular, the IBD relapses occurred with the same frequency in the two groups (Table 1). IBD exacerbations were documented in 13 out of 16 (81%) patients after SARS-CoV-2 infection, while in three patients, IBD flare-up occurred before SARS-CoV-2 infection and persisted even after infection resolution. Twelve out of 53 patients were receiving biologic agents (11 treated with anti-TNF and one with vedolizumab) before SARS-CoV-2 infection with no difference between the two groups. No patient was receiving combo-therapy. After SARS-CoV-2 infection resolution, only two patients needed pharmacological changes due to the IBD activity (one patient was treated with a higher dose of anti-TNF and the other one was treated with ustekinumab). Univariate analysis with logistic regression showed that female gender was a risk factor for Long COVID (3.33, 1.05–10.58, *p* = 0.041) while IBD duration appeared as a protective factor (0.37, 0.028–0.795, *p* = 0.037). However, multivariate analysis with multinomial logistic regression did not confirm such associations (Table 3). 

## 4. Discussion

A variety of cohorts and electronic medical record-based studies indicate that approximately 10–40% of SARS-CoV-2-infected patients can experience several physical and psychological symptoms that continue after the resolution of the acute phase. This condition, defined as Long COVID, is now recognized as a public health priority and its negative impact on the quality of life of the patients could be even more relevant in individuals with coexisting and debilitating pathologies [8,9]. Our study was undertaken to assess the frequency of Long COVID in IBD, as individuals living with IBD are already at risk of experiencing higher levels of psychological distress and poorer quality of life [14,15]. 

Our data indicate that Long COVID occurs in 40% of IBD population with no significant difference between CD and UC. As pointed out above, these data are consistent with the frequency of Long COVID in the general population, thus reinforcing the concept that IBD by itself does not represent a condition facilitating SARS-CoV-2 infection [16,17,18]. Asthenia was the most frequent symptom, as it was documented in 13 out of 21 patients with Long COVID. Although IBD patients can complain of asthenia, particularly during the active phases of the disease, we feel such a symptom was linked to SARS-CoV-2 infection. Indeed, the appearance of asthenia coincided with the onset of SARS-CoV-2 infection in 6 out of 13 patients while in the remaining seven cases, the symptom appeared just after the resolution of acute infection in individuals with no clinical relapse of IBD. Among patients with a previous history of SARS-CoV-2 infection, Long COVID was more frequent in females, thus confirming data of studies in the general population [8,9]. No difference was seen between individuals with Long COVID and those experiencing no symptoms in terms of current IBD therapy. Overall, these findings strongly suggest that IBD patients should be encouraged to continue their treatment during and after SARS-CoV-2 infection in order to prevent disease flares and IBD-associated complications [19]. 

To our knowledge, this is the first study documenting the frequency of Long COVID in IBD. Although such a frequency resembles that seen in the general population and does not seem to affect IBD course, we advise IBD patients to adhere to the vaccination campaign in order to reduce the risk of acute COVID-19 and its long-term complications.

## Figures and Tables

**Table 1:** Clinical and demographic characteristics of inflammatory bowel disease patients with a history of SARS-CoV-2 infection who either developed or not symptoms/signs suggestive of Long COVID.

Characteristics	Long COVID	No Long COVID	*p*
	(*n* = 21)	(*n* = 32)	
Female gender, *n* (%)	14 (67)	12 (38)	0.038
Age (y), median (range)	43 (21–68)	43 (21–74)	0.432
IBD diagnosis, *n* (%)			
CD	13 (62)	18 (56)	0.683
UC	8 (38)	14 (44)	0.578
IBD duration, years, median [range]	13 (3–24)	12.5 (3–37)	0.986
IBD therapy, *n* (%)			
Mesalamine	11 (52)	21 (66)	0.682
Biologic therapy	6 (29)	6 (19)	0.349
Antibiotic therapy	2 (10)	1 (3)	-
Immunosuppressive therapy	1 (5)	1 (3)	-
Steroid therapy	1 (5)	2 (6)	-
Experimental therapy	0	1 (3)	-
None	0	2 (6)	-
Duration COVID-19 infection, days, median [range]	15 (7–29)	15 (3–58)	0.154
Hospitalization for COVID-19, *n* (%)	2 (9)	2 (6)	-
COVID-19 symptoms, *n* (%)			
Fever	16 (76)	19 (59)	0.206
Asthenia	6 (29)	3 (9)	0.069
Neurologic symptoms (headache, anosmia, ageusia)	14 (67)	15 (47)	0.183
Respiratory symptoms (cough, dyspnoea, sore throat)	8 (38)	18 (56)	0.13
Digestive symptoms (diarrhea)	3 (14)	2 (6)	0.745
Musculoskeletal symptoms (myalgia)	5 (24)	11 (34)	0.938
Asymptomatic	0	4 (13)	0.092
Dermatological symptoms (rash)	1 (5)	0	-
Death for COVID-19, *n* (%)	0	0	-
Vaccine, *n* (%)	16 (76)	28 (88)	0.283
Type of vaccine, *n* (%)			
Pfizer	14/16 (88)	25/28 (89)	0.858
Moderna	2/16 (12)	2/28 (7)	0.552
AstraZeneca	0/16	1/28 (4)	0.444
IBD relapse post-COVID-19, *n* (%)	8 (38)	8 (25)	0.31

CD = Crohn’s disease, UC = ulcerative colitis.

**Table 2:** Type and duration of Long COVID symptoms in 21 IBD patients (*n* = 21).

Symptoms	Type, *n* (%)	Duration (Months), Median [Range]
Asthenia	13 (62)	6 (1–11)
Neurologic symptoms (anosmia, ageusia, memory loss)	8 (38)	3.5 (1–11)
Musculoskeletal symptoms (myalgia)	6 (29)	5 (2–12)
Dermatological symptoms (rash and hair loss)	8 (38)	2.5 (1–11)
Respiratory symptoms (dyspnoea)	3 (14)	6 (3–7)
Psychiatric symptoms (depression)	1 (5)	9

**Table 3:** Univariate analysis with logistic regression and multivariate analysis with multinomial logistic regression of risk factors for long-COVID. CD = Crohn’s disease.

	Univariate Analysis		Multivariate Analysis
	OR, 95% CI	*p* Value	*p* Value
Female gender (yes/not)	3.33, 1.05–10.58	0.041	0.244
Age (years)	1.02, 0.971–1.067	0.467	−
CD (yes/not)	1.19, 0.362–3.908	0.774	−
IBD duration, years	0.37, 0.028–0.795	0.037	0.996
Anti-TNF (yes/not)	1.44, 0.376–5.551	0.592	−
Mesalamine (yes/not)	0.786, 0.248–2.492	0.682	−
COVID-19 duration (years)	0.951, 0.886–1.021	0.164	−

## Data Availability

The data that support the findings of this study are available from the corresponding author (G.M.), upon reasonable request.
